# Contribution of aerobic and anaerobic capacity to 2000 m rowing performance

**DOI:** 10.1186/2052-1847-7-S1-P1

**Published:** 2015-08-11

**Authors:** Yusuke Shirai, Mikio Hiura, Yoshiraru Nabekura

**Affiliations:** 1 Graduate School of Comprehensive Human Sciences, University of Tsukuba, 1-1-1 Tennodai, Tsukuba-shi, Ibaraki, 305-8574, Japan; 2 Faculty of Sports and Health Studies, Hosei University, 4342 Aihara-machi, Machida-shi, Tokyo, 194-0298, Japan; 3 Fraculty of Health and Sport Science, University of Tsukuba, 1-1-1 Tennodai, Tsukuba-shi, Ibaraki, 305-8574, Japan

## Background

Previous studies strongly have supported importance of aerobic capacity for 2000m rowing performance [1-3] and there are few studies that demonstrated anaerobic capacity had critical role in rowing performance [4-6]. The purpose of the present study is to investigate the relationship between 2000m rowing performance and anaerobic capacity, which were estimated by critical power (CP) model [7,8] and by all-out tests of short duration as well. We also examined aerobic capacity.

## Subjects and Methods

Nine male collegiate rowers (age:20.0 ± 1.0 yrs, height:174.5 ± 4.5 cm，weight:70.1 ± 7.5 kg) performed 1) incremental exercise tests to determine VO_2max_, 2) CP test (400m, 600m, 800m and 1000m), and 3) 2000m test. For each subjects, the amount of work (power×time) was plotted against exercise time. The CP was determined as the slope of the linear regression between the work and time. The anaerobic work capacity (AWC) was determined as the y-intercept of the linear regression. AWC was evaluated with standard error of estimation (SEE) [8] for the sake of accurate observation. If SEE of regression line was greater than 10 % of AWC, it was recalculated except one trial that had largest error.

## Results

**Figure 1:**
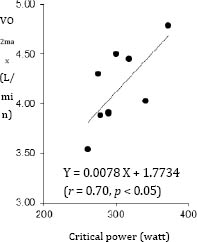
Relationship between maximal oxygen uptake and critical power.

**Figure 2:**
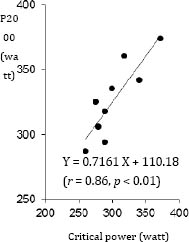
Relationship between critical power and P2000.

**Figure 3:**
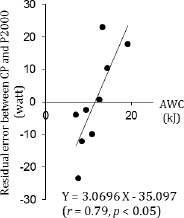
Relationship between AWC and residual error between CP and P2000.

CP (302.7 ± 35.2 watt) was correlated with VO_2max_ (4.1±0.4 L・min^-1^, *r* = 0.70, *p*<0.05, Figure 1) and power output during 2000 m test (P2000, 326.9 ± 29.3 watt, *r* = 0.86, *p*<0.01, Figure 2). AWC (11.4 ± 3.8 kJ) was not correlated with P2000 (*r* = 0.33). Our data demonstrated that there was significant correlation between AWC and residual error between CP and P2000 (*r* = 0.79, *p* < 0.01, Figure 3).

## Discussion

These results are in accordance with the established interpretation by which contribution of aerobic capacity to rowing performance are well recognized [1-6]. However, our data suggest that anaerobic capacity estimated by AWC also have a pivotal role for rowing performance. Since CP and AWC are affected by familiarity of subject to intensive exercise [8] and physiological condition such as fatigue caused by consecutive training sessions, examination of anaerobic capacity might predict rowing performance more precisely in practical competitive situation.

## References

[B1] Bourdin M Meissonier L Hager JP Lacour JR Peak power output predicts rowing ergometer performance in elite male rowers Int J Sports Med 2005 25 5 368 373 15241717 10.1055/s-2004-815844

[B2] Cosgrove MJ Wilson J Watt D Grant SF The relationship between selected physiological variables of rowers and rowing performance as determined by a 2000 m ergometer test J Sports Sci 1999 17 11 845 852 10.1080/026404199365407 10585164

[B3] Kramer JF Leger A Paterson DH Morrow A Rowing performance and selected descriptive, field, and laboratory variables Can J Appl Physiol 1999 19 2 174 184 8081321 10.1139/h94-013

[B4] Rowing Secher N Relly T, Secher N, Snell P, Williams C Physiology of sports 1990 London 259 285

[B5] Riechman SE Zoeller RF Balasekaran G Goss Fl Robertson RJ Prediction of 2000 m indoor rowing performance using a 30 s sprint and maximal oxygen uptake J Sports Sci 2002 20 9 681 687 12200919 10.1080/026404102320219383

[B6] Pripstein LP Rhodes EC McKenzie DC Coutts KD Aerobic and anaerobic energy during a 2-km race simulation in female rowers Eur J Appl Physiol Occup Physiol 1999 79 6 491 494 10.1007/s004210050542 10344457

[B7] Monodo RH Scherrer J The work capacity of synergic muscle group Ergonomics 1965 8 329 338 10.1080/00140136508930810

[B8] Hill DW The critical power concept: A review Sports Medicine 1991 16 4 237 254 8248682 10.2165/00007256-199316040-00003

